# Modulation of the Immune Response by Nematode Secreted Acetylcholinesterase Revealed by Heterologous Expression in *Trypanosoma musculi*

**DOI:** 10.1371/journal.ppat.1005998

**Published:** 2016-11-01

**Authors:** Rachel Vaux, Corinna Schnoeller, Rita Berkachy, Luke B. Roberts, Jana Hagen, Kleoniki Gounaris, Murray E. Selkirk

**Affiliations:** Department of Life Sciences, Imperial College London; University of Medicine & Dentistry New Jersey, UNITED STATES

## Abstract

Nematode parasites secrete molecules which regulate the mammalian immune system, but their genetic intractability is a major impediment to identifying and characterising the biological effects of these molecules. We describe here a novel system for heterologous expression of helminth secreted proteins in the natural parasite of mice, *Trypanosoma musculi*, which can be used to analyse putative immunomodulatory functions. Trypanosomes were engineered to express a secreted acetylcholinesterase from *Nippostrongylus brasiliensis*. Infection of mice with transgenic parasites expressing acetylcholinesterase resulted in truncated infection, with trypanosomes cleared early from the circulation. Analysis of cellular phenotypes indicated that exposure to acetylcholinesterase in vivo promoted classical activation of macrophages (M1), with elevated production of nitric oxide and lowered arginase activity. This most likely occurred due to the altered cytokine environment, as splenocytes from mice infected with *T*. *musculi* expressing acetylcholinesterase showed enhanced production of IFNγ and TNFα, with diminished IL-4, IL-13 and IL-5. These results suggest that one of the functions of nematode secreted acetylcholinesterase may be to alter the cytokine environment in order to inhibit development of M2 macrophages which are deleterious to parasite survival. Transgenic *T*. *musculi* represents a valuable new vehicle to screen for novel immunoregulatory proteins by extracellular delivery in vivo to the murine host.

## Author Summary

Parasitic nematodes are known to secrete proteins which suppress or divert the host immune response in order to promote their survival. However it has proven very difficult to delete or silence genes in order to decipher the function of the proteins they encode. We have developed a method whereby genes can be expressed in a live vehicle or carrier which is then used to infect mice, and the effects on the immune response can be determined. As proof of principle, we used this system to express a gene from a parasitic worm for an enzyme which hydrolyses acetylcholine, a signalling molecule which regulates a wide variety of physiological functions, including those of the immune system. Expression of this enzyme resulted in the carrier being cleared early from the circulation, and was associated with functional polarisation of macrophages away from a phenotype known to be deleterious to parasitic worms. We conclude that by doing this, the enzyme may act to promote parasite survival.

## Introduction

Helminth parasites have evolved sophisticated mechanisms to regulate and suppress host immune responses, thought to underlie the inverse relationship between infection and the incidence of inflammatory disorders [1] [2]. Molecules secreted by helminths induce these effects either directly or via induction of endogenous mechanisms for maintaining homeostasis in the host immune system [3]. Defining the parasite molecules which induce these effects has proven more difficult, requiring laborious purification or cloning, expression and testing individual proteins on a case-by-case basis. In addition to those known or suspected to have immunomodulatory properties, there exist a plethora of orphan proteins which have been demonstrated or predicted to be secreted by helminth parasites [3]. Many of these are likely to have regulatory effects on the host immune system, but the genetic intractability of helminth, and nematode parasites in particular, has made progress on this front very slow [4] [5]. The most commonly used method for gene silencing, RNA interference (RNAi), has proven difficult to employ in parasitic nematodes, primarily through problems with delivery and spread of dsRNA [6]. Heterologous expression of helminth parasite genes in a suitable vehicle, i.e. a gain of function approach, provides another means to interrogate the properties of individual gene products.

Many nematode parasites secrete acetylcholinesterases (AChEs), classically associated with terminating signalling by acetylcholine (ACh) at synapses and neuromuscular junctions. Previous hypotheses on the role of nematode secreted AChEs have focused on inhibition of host cholinergic signalling which might contribute to dislodging parasites from the gastrointestinal tract, such as smooth muscle contraction, mucus secretion by goblet cells, and fluid secretion by enterocytes [7]. More recently it has become apparent that cholinergic signalling influences the immune system. This was first identified by suppression of macrophage inflammatory cytokines such as TNFα, IL-1β and IL-18 [8], which was subsequently discovered to be effected by ACh released from CD4+ T cells [9]. B cells also release ACh which acts on endothelial cells to inhibit expression of integrins and thus suppress inflammatory extravasation of neutrophils [10]. In contrast to these anti-inflammatory effects of ACh on innate immunity, we recently showed that ACh acts as a co-stimulatory signalling molecule for CD4+ T cell activation and cytokine production [11]. Cholinergic signalling in relation to immunity is thus complex and multi-layered, and it is difficult to predict what effect parasite secreted AChEs might have in vivo. We have developed a vehicle which enables us to dissect the immunomodulatory roles of helminth secreted proteins, and used AChE from the intestinal nematode parasite *N*. *brasiliensis* as a test case.

*Trypanosoma musculi* is a natural parasite of mice which inhabits the bloodstream and extracellular tissue fluids of its host [12]. Infection normally lasts for approximately three weeks, before it is cleared from the peripheral circulation and extracellular fluids by an antibody–dependent, cell-mediated process [13] [14] [15]. The brevity of infection and relatively benign pathology make *T*. *musculi* an excellent vehicle in which to express potential immunoregulatory molecules, using infection of mice as an in vivo screen for effects on the immune system. We constructed plasmids designed to integrate into the *T*. *musculi* genome and direct secretion of proteins encoded by exogenous genes. Here, we use this vehicle to deliver *N*. *brasiliensis* AChE to the murine host, and demonstrate that this modulates the immune system via reduction of Th2 cytokines and influencing macrophage function.

## Results

### Propagation of *T*. *musculi* in vitro

Previous reports had indicated that *T*. *musculi* could be grown in medium conditioned by murine macrophages (adherent spleen cells) [16]. *T*. *musculi* Lincicome strain were used to infect mice, parasites isolated from peripheral blood and cultured under a range of conditions. Optimal growth was obtained by culture in 50% HMI-9 [17] / 50% conditioned medium from the mouse macrophage cell line J774 [18] or RAW 264.7 [19]. After 3 passages in this medium, cells grew rapidly to densities of 3 x 10^6^ ml^-1^ before requiring passage (S1 Fig). Blasticidin, puromycin and neomycin were all biocidal, killing *T*. *musculi* after four days, whereas hygromycin and phleomycin were biostatic (S1 Fig), indicating that the first 3 drugs could be used for selection of transfected parasites.

### Vector construction

**Fig 1:**
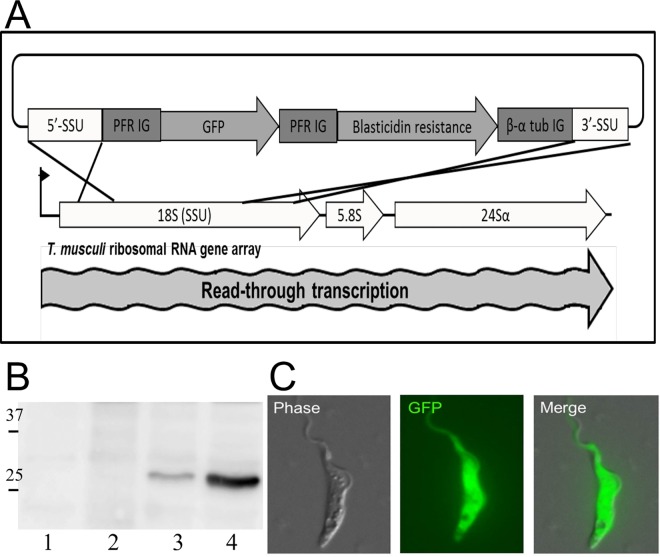
Generation of transgenic *T*. *musculi* and expression of GFP. (A) Schematic depiction of expression vector pSSUGFP, utilising 5´ and 3´ regions to integrate into the 18S SSU rRNA gene locus, and both PFR and β-α tubulin intergenic regions to effect RNA processing of eGFP and blasticidin resistance genes. (B) Detection of eGFP expression by western blot. Lane 1: Wild type *T*. *musculi*; Lane 2: Parasites transformed with expression cassette in which eGFP was incorporated into the tubulin gene array (pTubGFP); Lane 3: Expression cassette with eGFP incorporated into the SSU rRNA locus (pSSUT7GFP) in a cell line with T7 RNA polymerase incorporated into the tubulin array (pT7polyNeo); Lane 4: Expression cassette with eGFP incorporated into the SSU rRNA locus alone (pSSUGFP). Molecular mass markers are shown in kDa. See S2 Fig for different constructs. (C) Detection of eGFP expression by fluorescence microscopy: eGFP incorporated into the SSU rRNA locus.

*T*. *musculi*-specific expression vectors were generated based on a strategy employed for *Trypanosoma theileri* which takes advantage of read-through transcription at the 18S small subunit ribosomal RNA (SSU rRNA) gene locus [20]. An expression cassette containing a drug-selection gene was designed to integrate into the SSU rRNA locus by homologous recombination, using sequences from the intergenic regions (IR) of *T*. *musculi* paraflagellar rod (PFR) and tubulin genes to effect RNA processing and capping with the 5´ spliced leader sequence. The only gene from *T*. *musculi* sequenced at the time of the study was the SSU rRNA gene [21]. Primers were made to this, and to consensus sequences for tubulin and paraflagellar rod (PFR) genes from other trypanosome species, and the α-β tubulin IR, the β-α tubulin IR and the PFR IR were isolated from *T*. *musculi* by polymerase chain reaction (PCR) to facilitate construction of the expression cassette (Fig 1A). In order to test if this vector could direct expression of exogenous genes, we inserted the gene for eGFP into the expression cassette, linearised it and transfected *T*. *musculi* by electroporation, selecting transfectants with blasticidin. Incorporation of eGFP into the SSU rRNA locus was confirmed by linking PCR, and expression confirmed by western blot (Fig 1B) and fluorescence microscopy (Fig 1C). Initially, we constructed another vector which inserted eGFP into the tubulin array (S2C Fig), but expression was relatively poor. Much higher expression of eGFP was achieved when placed in the SSU rRNA locus, and this was not enhanced by incorporation of T7 polymerase promoters and terminators, in concert with insertion of the T7 polymerase gene into the tubulin array, a strategy which has been used successfully to boost transcription in *T*. *brucei* [22] (Fig 1B and S2A and S2B Fig).

Because we aimed to use the vehicle to express immunomodulatory secreted proteins, we made an additional cassette with sequences for the N-terminal signal peptide of the *T*. *musculi* homologue of BiP/GRP78 (N-BiP) to direct secretion via the endoplasmic reticulum, again using degenerate primers to conserved regions of the BiP/GRP78 gene from other trypanosome species, extending by 5´ RACE and amplifying by PCR to isolate relevant sequences, then placing them in the cassette between the PFR intergenic regions immediately upstream of the target gene cloning site (S2E Fig).

### Expression of *N*. *brasiliensis* AChE B

**Fig 2:**
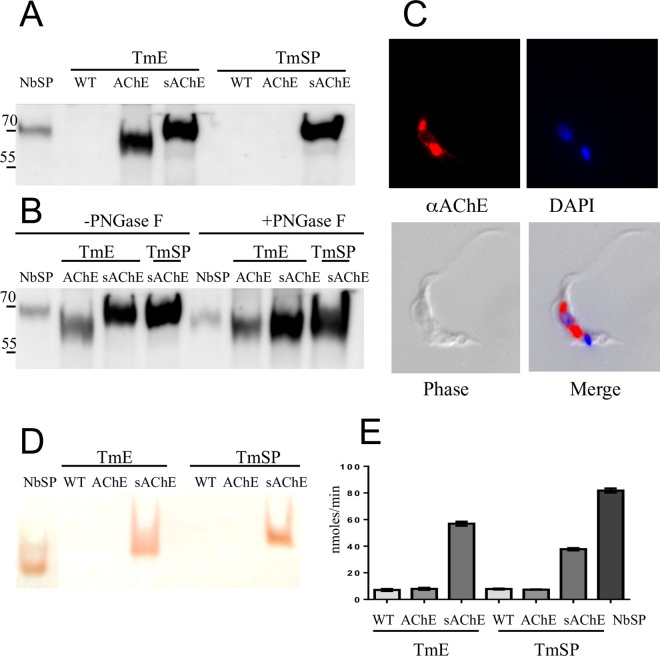
Expression of *N*. *brasiliensis* AChE B in *T*. *musculi*. (A) Detection by western blot. NbSP: Secreted products from *N*. *brasiliensis*; TmE: *T*. *musculi* extracts; TmSP: *T*. *musculi* secreted products. WT: Wild type trypanosomes; AChE; *T*. *musculi* expressing cytosolic AChE; sAChE: *T*. *musculi* expressing secreted AChE. (B) Tm-sAChE is glycosylated. Extracts and secreted products as in A), either with (+) or without (-) PNGase F treatment. Molecular mass markers are shown in kDa. (C) Tm-sAChE stained with antibody to *N*. *brasiliensis* AChE B and DAPI and viewed by indirect immunofluorescence. (D) Visualisation of AChE activity after non-denaturing gel electrophoresis, abbreviations as in panel A. (E) AChE activity measured by Ellman assay, abbreviations as in panel A. TmE: *T*. *musculi* extracts from 5 x 10^5^ trypanosomes; TmSP: *T*. *musculi* secreted products from 5 x 10^4^ trypanosomes cultured for 24 hrs. Data are shown as the mean ±SEM, assayed in triplicate.

In order to express *N*. *brasiliensis* AChE B [23] in *T*. *musculi*, we inserted the coding sequence for the mature protein (minus the signal peptide) into both the cytosolic and the secretory N-BiP vector (S2D and S2E Fig), selecting transformants by antibiotic resistance and confirming insertion by linking PCR. AChE was abundantly expressed by both vectors and detected by western blot in trypanosome extracts, but was only detectable in secreted products of parasites transformed with the N-BiP vector (Fig 2A). AChE B expressed by the latter vector had a higher mass (67 kDa) than that expressed by the cytosolic vector (62 kDa) due to N-linked glycosylation (Fig 2B), indicating that it was being trafficked though the endoplasmic reticulum and released via a conventional secretory pathway, and localisation by immunofluorescence was consistent with this interpretation (Fig 2C). As there is a simple gel-based activity assay for AChE [24], we tested whether recombinant proteins were enzymatically active. Fig 2D shows that active AChE was detected in both extracts and secreted products of trypanosomes transfected with the N-BiP construct (Tm-sAChE), but that AChE expressed without the *T*. *musculi* signal peptide (Tm-AChE) had no demonstrable enzymatic activity, even in parasite extracts which contained high levels of recombinant protein. This was confirmed by Ellman assay [25] (Fig 2E).

### Survival of transgenic *T*. *musculi* in vitro and in vivo

**Fig 3:**
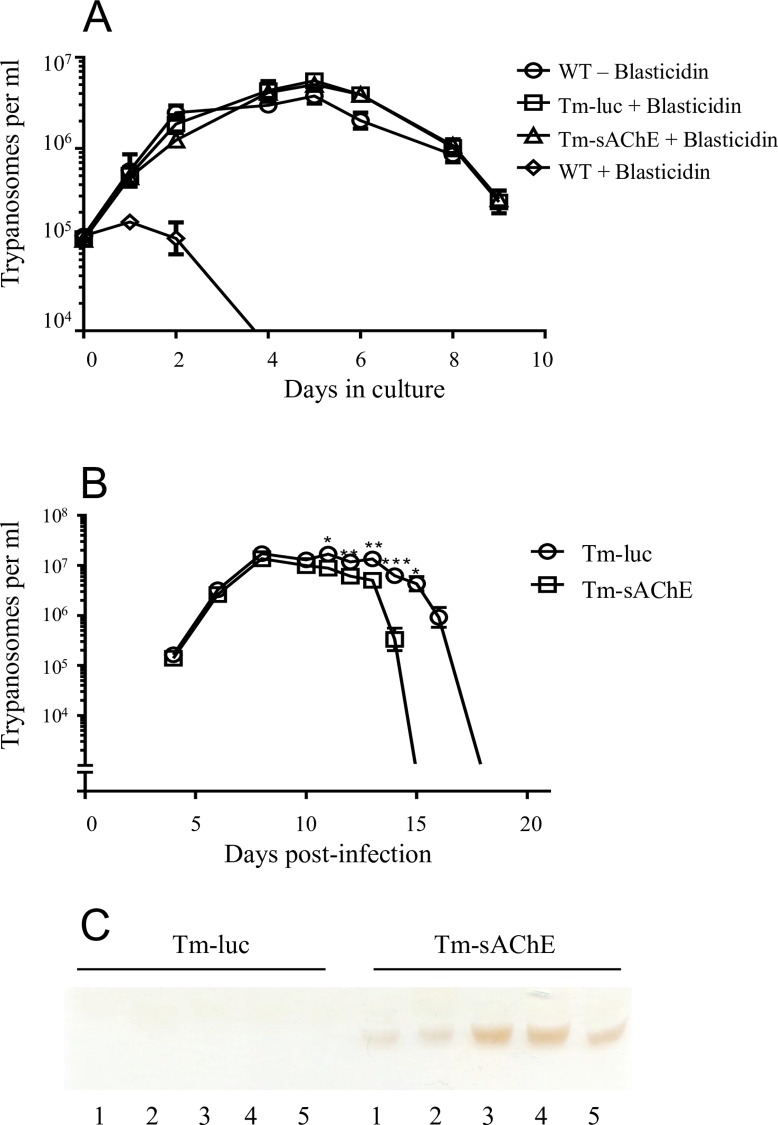
Growth and survival of transgenic *T*. *musculi* in vitro and in vivo. (A) Growth *in vitro* of wild type *T*. *musculi* (WT), those engineered to express secreted AChE (Tm-sAChE) and expressing cytosolic luciferase (Tm-luc). (B) Survival of Tm-sAChE compared to Tm-luc *in vivo*. Parasitaemia in peripheral blood monitored over the course of infection of female BALB/c mice. Data are shown as the mean ±SEM (n = 5) and are representative of three independent experiments. *p<0.05, **p<0.01, ***p<0.001. (C). Detection of AChE in serum of mice 8 days post-infection with Tm-luc and Tm-sAChE by activity-based gel assay [23]. Samples of serum (5 μl) from individual mice were loaded in each lane.

The growth in vitro of Tm-sAChE was compared to wild type parasites (Tm) and those engineered to express cytosolic luciferase (Tm-luc), and observed to exhibit no significant difference (Fig 3A). We next infected female BALB/c mice by intraperitoneal (ip) inoculation of 2 x 10^5^ parasites. Tm-luc were observed to follow an identical course of parasitaemia to wild type parasites, and were thus used as a standard control. A striking difference in the course of parasitaemia was observed with Tm-sAChE however. Tm-luc exhibited a typical pattern of infection in which the parasitaemia in peripheral blood reached a peak by day 8, remained relatively constant for 5 days, with subsequent progressive clearance from day 14 such that all parasites were eliminated by day 18. In contrast, whilst Tm-sAChE reached an identical peak parasitaemia with the same kinetics, parasites were then abruptly cleared from the circulation 3 days before their normal counterparts (Fig 3B). AChE secreted by *T*. *musculi* was readily detectable in plasma taken from mice during peak parasitaemia (day 8) by activity-based gel assay (Fig 3C).

**Fig 4:**
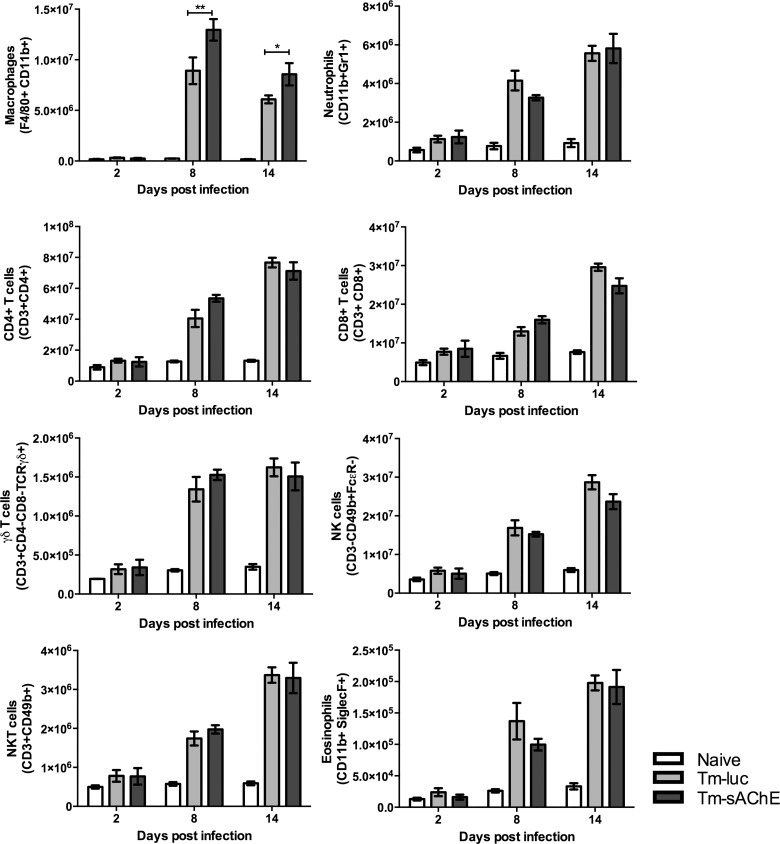
Cellularity of spleens from mice infected with transgenic *T*. *musculi*. Phenotyping was performed by flow cytometry as described in Materials and Methods. Data are shown as the mean ± 1SEM (n = 5) and are representative of two independent experiments with 5 mice in each group. *p<0.05, **p<0.01. White bar = uninfected mice; Light grey bar = mice infected with Tm-luc; Dark grey bar = mice infected with Tm-sAChE.

**Fig 5:**
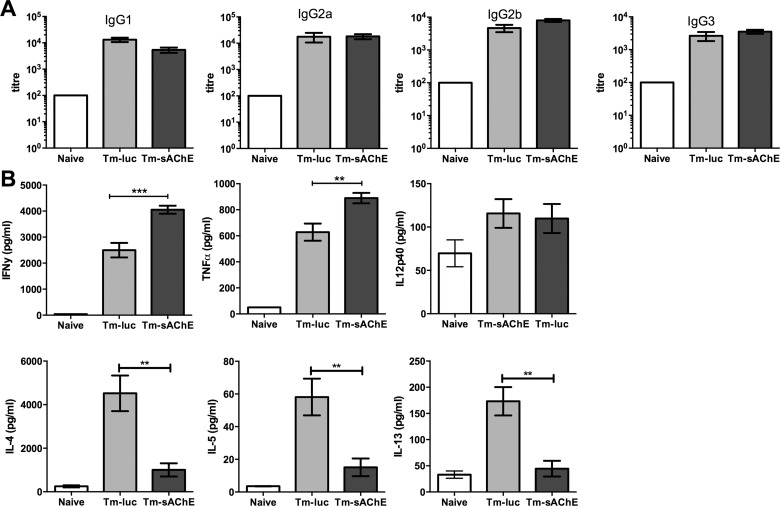
Altered immune responses in mice infected with Tm-sAChE. (A) Specific antibody responses (end-point titres) to *T*. *musculi* at d14 post-infection. (B) Cytokine responses from splenocytes at d14 post-infection. Data are shown as the mean ±SEM (n = 5) and are representative of three independent experiments. **p<0.01, ***p<0.001. White bar = uninfected mice; Light grey bar = mice infected with Tm-luc; Dark grey bar = mice infected with Tm-sAChE.

All leukocyte populations examined were expanded in the spleens of infected mice. There was little reproducible difference between mice infected with Tm-sAChE and Tm-luc, although higher numbers of splenic macrophages (F4/80+ CD11b+) were observed in mice infected with Tm-sAChE (Fig 4). There was no significant difference in IgG subclass antibody titres during the elimination phase between mice infected with either parasite (Fig 5A). Significantly however, splenocytes from mice infected with Tm-sAChE showed enhanced IFNγ and TNFα production during the elimination phase, accompanied by lower levels of IL-4, IL-13 and IL-5 (Fig 5B).

**Fig 6:**
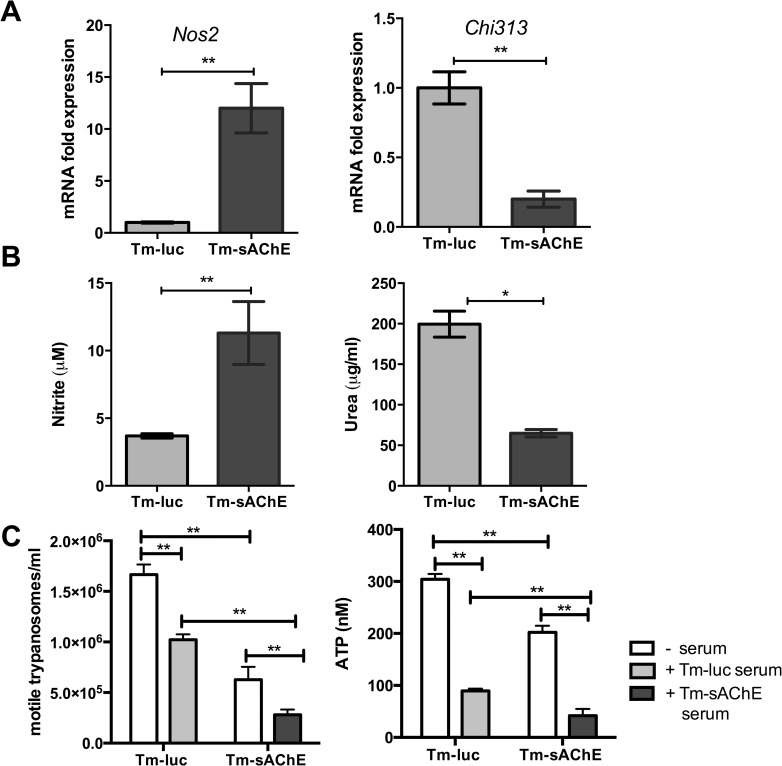
**Macrophage phenotype and cytotoxicity** (A) Expression of *Nos2* and *Chi3l3* (*Ym1*) in peritoneal macrophages isolated from mice infected with Tm-luc and Tm-sAChE at d14 post infection was determined by qPCR and is shown as expression relative to the median of mice infected with Tm-luc. Light grey bar = mice infected with Tm-luc; dark grey bar = mice infected with Tm-sAChE. (B) Nitric oxide synthase and arginase activity assayed via production of nitrite and urea by peritoneal macrophages isolated from mice infected with Tm-luc and Tm-sAChE at d14, stimulated with 10 μg ml^-1^ *T*. *musculi* extract. (C) Cytotoxicity of macrophages isolated from mice at d14 post-infection with Tm-luc or Tm-sAChE towards wild-type *T*. *musculi*, in the absence or presence of serum. Parasite viability was assayed by counting the number of motile trypanosomes, and also determined by ATP assay. Data are shown as the mean ±SEM (n = 3), and are representative of two independent experiments. *p<0.05, **p<0.01.

Macrophages isolated from the peritoneum of mice infected with Tm-sAChE at the time of parasite clearance showed higher levels of expression of *Nos2* and lower levels of *Chi3l3* (Ym1) compared to controls infected with Tm-luc (Fig 6A). In addition, when stimulated with *T*. *musculi* extract (TmE), macrophages from Tm-sAChE-infected mice produced elevated levels of nitric oxide (which was below the limits of detection in mice infected with Tm-luc) and lower arginase activity (Fig 6B). In order to determine their toxicity towards trypanosomes, we tested them in killing assays. In the absence of immune serum, macrophages from mice infected with Tm-sAChE showed greater cytotoxicity against parasites than macrophages from mice infected with Tm-luc, and this was enhanced by immune serum (Fig 6C).

## Discussion

Heterologous expression of helminth parasite genes in a suitable vehicle provides a means to examine possible effector functions, with survival and replication of the vehicle in vivo as one readout for immunomodulatory activities which makes no assumptions on the specific function or pathway targeted. The only other study thus far which examined heterologous expression as a means of dissecting parasitic helminth gene function in vivo used *Leishmania mexicana* as a vehicle for expression of *Brugia malayi* ALT proteins [26] [27]. Profound effects were described on host macrophage gene expression, the ensuing immune response, and parasite replication. However, as *L*. *mexicana* is an intracellular parasite and helminths are predominantly extracellular, delivery by this vehicle may not be suitable for analysing the effects of many helminth secreted proteins. We selected *T*. *musculi* due to the fact that it is a natural extracellular parasite of mice which is well tolerated despite reaching very high parasitaemia.

We have demonstrated that our plasmids are integrated into the *T*. *musculi* genome, and that they direct expression of GFP and enzymatically active *N*. *brasiliensis* AChE B. Other heterologous gene expression systems involving *Trypanosoma spp*. use either known promoter sequences from that species, read-through transcription, or a T7 RNA polymerase based-system [22] [20]. No promoter sequences are known for *T*. *musculi*, so we tested the latter two approaches, following a strategy employed for *T*. *theileri* [20]. The *N*. *brasiliensis* AChE B gene was expressed at a high level when placed in the SSU rRNA locus, and thus we adopted this as our standard vector for delivery of exogenous gene products. The rationale for isolating *T*. *musculi* intergenic sequences to effect RNA processing was based on the unpredictable cross-functionality of processing signals between trypanosomatid species [28] as noted by Mott *et al*. [20].

Interestingly, expression of AChE B without a signal peptide resulted in no detectable enzymatic activity. This is most likely due to the fact that the conformation of AChEs is highly dependent on 3 intramolecular disulphide bonds [29], and that unless the enzyme is translocated into the endoplasmic reticulum, it is unlikely to fold correctly. This makes it difficult to quantitate expression levels with or without the signal peptide, although western blots suggest that there was little difference (Fig 2).

The design of our vehicle relied heavily on that successfully employed to express exogenous genes in *T*. *theileri*, a natural, non-pathogenic parasite of bovids [20]. The rationale for the former work was to develop a vehicle suitable for delivery of live vaccines against pathogens of cattle, in addition to other proteins of therapeutic use. *T*. *theileri* and *T*. *musculi* are stercorarian trypanosomes, transmitted via faeces of tabanid flies and fleas respectively, generally through the oral mucosa. *T theileri* lives in extracellular fluids at very low levels (about 10^2^ ml^-1^) for the lifetime of the bovid host, whereas *T*. *musculi* reaches very high levels of parasitaemia (over 10^8^ ml^-1^ in peripheral blood) for about 3 weeks. Following clearance from the peripheral circulation, *T*. *musculi* persists in the murine host as a small population (1–5 x 10^4^) in the vasa recta of the kidneys [30], thought to result from the hypertonic conditions in this site which preclude antibody binding [31]. *T*. *theileri* therefore offers a persistent antigenic stimulus suitable for vaccine delivery, whereas the high parasitaemias reached by *T*. *musculi* allow delivery of substantial amounts of protein over a relatively short time, suitable for interrogating how an exogenous protein might suppress or skew the host immune response. This was evident in the current study, in which high levels of nematode AChE were detectable in the bloodstream (Fig 3). *N*. *brasiliensis* AChE B is an extremely stable protein [32]. This stability, combined with its fast catalytic activity [23], makes the enzyme well adapted to operate efficiently in the mammalian host. *N*. *brasiliensis* secrete three discrete AChEs, with indistinguishable substrate specificities and very similar catalytic properties [7]. AChE B was selected as a representative example for this study, but we assume the enzymes perform the same function.

Cholinergic signalling suppresses expression of inflammatory cytokines by macrophages via α7 nicotinic receptors (nα7R) [33], whereas ligation of the m3 muscarinic receptor (m3R) on CD4+ T cells potentiates cellular activation and cytokine production [11]. It was thus unclear what effect secreted AChEs might have on the immune response to an infectious agent. Macrophages from mice infected with Tm-sAChE showed elements of polarisation to an M1 phenotype, with elevated production of nitric oxide and lower arginase activity. T cell numbers and cytokine responses were intact in mice infected with Tm-sAChE, although the latter were skewed towards a Th1 profile. These data suggest that hydrolysis of ACh by AChE introduced by transgenic *T*. *musculi* has a more pronounced effect on the macrophage cholinergic anti-inflammatory pathway than inhibition of co-stimulatory signals on T cells. Consistent with this interpretation, recent data indicate that AChEs expressed on leukocytes play a role in regulating inflammatory cytokine production: intraperitoneal administration of LPS to mice leads to a systemic reduction in AChE activity, and exposure of splenocytes in vitro to LPS results in substantial reduction of AChE expression, suggesting that this is part of the reflex response to maximise the anti-inflammatory capacity of ACh [34]. Down-regulation of AChE expression is effected by microRNA (mir)-132, and transgenic mice over-expressing 3´-UTR null AChE (i.e. unresponsive to regulation by mir-132) showed excessive production of inflammatory cytokines by macrophages and an impaired cholinergic anti-inflammatory response [34]. Somewhat different results were obtained in a recent study which examined the response to *Citrobacter rodentium* in m3R-/- mice. Contrasting effects of cholinergic signalling through the nα7R and the m3R on macrophage function were described, with macrophages polarised to either an M1 or an M2 phenotype depending on the cytokine environment [35]. Nevertheless, in our current system, the net effect of over-expressing nematode AChE during systemic infection of mice with our vehicle is polarisation to a Th1 environment and skewing of macrophages to an M1 phenotype. Other recent published work is consistent with this observation, as cholinergic stimulation of dendritic cells promotes production of IL-4, IL-5 and IL-13 in the course of a mixed lymphocyte reaction [36].

We conclude that the alterations in parasitaemia brought about by expression of nematode secreted AChE in *T*. *musculi* are largely effected by the opposing roles of cytokines and macrophages in immunity to trypanosomatids and helminths. Immunity to *T*. *brucei* is IFNγ-dependent and largely Th1-mediated [37]. Toll-like receptor (TLR)-4 has been described to be an important determinant of immunity to *T*. *cruzi*, and has been linked to optimal production of IFNγ, TNFα and production of nitric oxide [38]. Although less is known about immunity to *T*. *musculi*, it is well established that C3H/HeJ mice are by far the most susceptible mouse strain, harbouring extremely high parasitaemia [39], most likely due to mutation of the TLR-4 gene [40]. Glycoinositolphospholipids (GIPLs) from *T*. *cruzi* are ligands for TLR-4 signalling in mice [41], and it is likely that *T*. *musculi* express similar ligands, as macrophages from infected mice produced nitric oxide when stimulated with parasite extracts (Fig 6B).

*T*. *musculi* are cleared by an antibody–dependent, cell-mediated mechanism, with curative antibodies produced late in the second week of infection [12] [15]. IgG2a appears to be the principal antibody isotype associated with clearance [13], although IgG1 and IgG2b have also been implicated [14] [42]. In the current study, we observed no significant difference in titres of IgG subclasses between mice infected with Tm-sAChE and Tm-luc at the point of clearance. Peritoneal macrophages from *T*. *musculi*-infected mice exhibit trypanostatic activity from day 10 onwards, and this activity was observed to be maximal at day 14 post-infection [43]. Antibody-facilitated killing by macrophages is enhanced by IFNγ and found to be due in part to production of nitric oxide [15], consistent with our observation of optimal killing by macrophages from mice infected with Tm-sAChE (Fig 6C).

In contrast, there is now a substantial body of literature implicating M2 macrophages in immunity against nematode parasites, acting in concert with type 2 cytokines produced by CD4+ T cells, innate lymphoid cells (ILC2s), and/or antibodies [44] [45] [46]. Our current data suggest that one of the functions of nematode secreted acetylcholinesterase may be to alter the cytokine environment in order to suppress development of M2 macrophages and thus promote parasite survival. In the case of *N*. *brasiliensis*, this could occur either in the lungs or the GI tract, as expression of the enzyme is activated by the transition to parasitism in third stage larvae, and it is expressed at high levels in both L4 and adult worms.

## Materials and Methods

### Ethics Statement

This study was approved by the Animal Welfare Ethical Review Board at Imperial College London, and was licensed by and performed under the UK Home Office Animals (Scientific Procedures) Act Personal Project Licence number 70/8193: ‘Immunomodulation by helminth parasites’.

### Cell culture

Macrophage-conditioned medium was prepared from RAW 264.7 murine macrophages maintained in Dulbecco’s Modified Eagle’s Medium (DMEM) at 37°C, 10% foetal calf serum (FCS), 2 mM L-glutamine, 100 units ml^-1^ penicillin and 100 μg ml^-1^ streptomycin. Cells were grown to approximately 80% confluence, the supernatant harvested, centrifuged at 300 x *g* for 6 min and passed through a 0.2 μm filter. *T*. *musculi* were cultured at 37°C in 5% CO_2_ in medium containing 50% modified HMI-9 [17] containing 10% FCS and 50 μM mercaptoethanol, and 50% macrophage-conditioned medium. Transgenic *T*. *musculi* cell lines were maintained in 15 μg ml^-1^ blasticidin or 2 μg ml^-1^ neomycin.

### Parasite infection

*T*. *musculi* Lincicome strain were obtained from American Type Culture Collection (ATCC). Female BALB/c mice (6–8 weeks) were routinely infected by intraperitoneal inoculation of 2 x 10^5^ parasites. Parasitaemia was monitored by tail snip, red blood cells lysed in 0.83% ammonium chloride and parasites counted in a haemocytometer [39].

### Generation of expression vectors and recombinant cell lines

Vectors were constructed from pUC19 plasmid, with eGFP and blasticidin resistance genes isolated from pMCGFPtubBlast [47]. Consensus sequences for α tubulin, β tubulin and paraflagellar rod (PFR) genes from other trypanosome species were generated using the program GeneFisher2 (Bielefeld University) and sequences from NCBI nucleotide and TriTryp databases. Degenerate primers for consensus sequences were used to amplify the α-β tubulin IR, the β-α tubulin IR and the PFR IR by PCR from genomic DNA of *T*. *musculi*, which were then inserted into the plasmid using appropriate restriction sites. The plasmid was modified with 5´ and 3´ fragments of the *T*. *musculi* small subunit ribosomal RNA (SSU rRNA) gene to generate a vector for insertion into the locus by homologous recombination. This vector was modified to incorporate sequences for the N-terminal signal peptide of the *T*. *musculi* homologue of BiP/GRP78 (N-BiP) to direct secretion via the endoplasmic reticulum. Degenerate primers, based on alignment of *T*. *brucei* BiP and *T*. *cruzi* hsp 70 sequences, were used to amplify a DNA fragment for *T*. *musculi* BiP, which was extended by 5´ RACE, and sequences encoding the signal peptide and cleavage site identified by SignalP 4.0 [48]. For the generation of transgenic *T*. *musculi* expressing *N*. *brasiliensis* AChE B [23], the eGFP gene was replaced with cDNA sequence for mature AChE B, with or without the BiP signal peptide sequence. Click beetle red luciferase (*CBRLuc*) was inserted without the signal peptide sequence. After sequencing to confirm the correct insertion and reading frame of the relevant fragments, plasmids were cut with BamHI, AlwNI or ScaI to linearise the cassette for incorporation into the *T*. *musculi* genome. Approximately 10 μg of DNA was used to transfect 3 x 10^7^ wild type cells from a *T*. *musculi* culture growing in logarithmic phase using the Amaxa Nucleofector kit (Lonza) using conditions described by Burkard *et al*. [49]. Cells were transferred to pre-warmed medium and cultured for 24 hrs at 37°C, 5% CO_2_, prior to addition of 15 μg ml^-1^ blasticidin or 2 μg ml^-1^ neomycin to select transfectants. Clonal cell lines were derived by serial dilution and subsequent expansion.

### Immunoblotting

*T*. *musculi* were incubated in lysis buffer (50 mM HEPES pH 7.5, 10% glycerol, 1% Triton X-100, 1.5 mM MgCl2,1 mM EGTA) at room temperature for 20 minutes, then boiled in SDS loading buffer for 5 minutes. A total of 5 x 10^5^ cells were loaded per well on 12% polyacrylamide gels, western blotting performed and signals detected by chemiluminescence according to standard procedures. In some cases protein samples were deglycosylated with PNGase F (New England Biolabs) under standard conditions prior to addition of loading buffer. The antibodies used were polyclonal rabbit anti-GFP (VWR) diluted 1:1,000 and polyclonal rabbit anti-AChE B [23] diluted 1:500, followed by HRP-conjugated secondary antibody.

## Acetylcholinesterase Activity

AChE activity was determined as previously described by Ellman assay [50]. The standard incubation mixture contained 1 mM acetylthiocholine (ASCh) iodide as substrate in the presence of 1 mM 5,5’-dithiobis(2-nitrobenzoic acid) (DTNB) in 100 mM sodium phosphate pH 7.0 at 20°C. Reactions were monitored by measuring the absorbance at 412 nm, and hydrolysis of ASCh calculated from the extinction coefficient of DTNB. AChE activity in mouse serum, and extracts and secreted products of *T*. *musculi*, was also visualized following resolution on an 8% gel under non-denaturing conditions according to Karnovsky and Roots [24].

### Cytokine ELISA, nitric oxide and arginase assay

Cytokine ELISAs were performed using coating and biotinylated detection antibodies from BioLegend, with streptavidin-conjugated HRP for detection. Cells were recovered from the spleens of infected or control mice, plated at 2 x 10^6^ ml^-1^ in 96 well plates, stimulated with either 25 ng ml^-1^ phorbol 12-myristate 13-acetate (PMA)/250 ng ml^-1^ ionomycin, or 10 μg ml^-1^ anti-CD3/anti-CD28 for 24 hrs, and supernatants removed for assay. Nitric oxide production was determined by Griess assay (Promega) following incubation of peritoneal macrophages for 24 hrs in Dulbecco’s Modified Eagle's Medium (DMEM), stimulated with 10 μg ml^-1^ *T*. *musculi* extract. Arginase activity was determined by production of urea as described by Pesce *et al*. [51] following stimulation with *T*. *musculi* extract and incubation as above. Briefly, macrophages were lysed with 0.1% Triton X-100 supplemented with protease inhibitors, incubated in 50 μl of 10 mM MnCl_2_, 50 mM Tris HCl (pH 7.5) at 55°C for 10 min to activate the enzyme, then 25 μl incubated for one hr at 37°C with an equal volume of 500 mM L-Arginine (pH 9.7). The reaction was stopped using 400 μl of acid mixture (H_2_SO_4_:H_3_PO_4_:H_2_O at a ratio of 1:3:7). Thereafter, the colourimetric reaction was initialised by adding 25 ul of 9% α-isonitrosopropiophenone and incubated at 100°C for 45 min, followed by 10 min in the dark at room temp. Absorbance was measured at 450 nm, and converted to urea concentration by comparison with a standard curve.

### Antibody ELISA

Parasite-specific antibodies were measured by ELISA, coating plates with WT *T*. *musculi* extract at 5 ug ml^-1^ in 0.1 M carbonate buffer. Sera from infected mice were serially diluted in phosphate buffered saline (PBS) 1% bovine serum albumin (BSA), and end-point titres determined at background binding. HRP-conjugated goat anti-mouse Ig subtypes were diluted 1:2,000 according to manufacturers’ instructions, and reactions visualised with 3,3’,5,5’-Tetramethylbenzidine.

### Immunofluorescence

Cells were washed in PBS, fixed in 2% paraformaldehyde for 15 minutes at room temperature, quenched in PBS/0.1% glycine for 10 minutes and blocked with PBS/2% FCS for 30 minutes. Staining was effected with anti-AChE B for 1 hour in PBS/2% FCS. Cells were washed 3x in PBS, incubated with FITC-labelled secondary antibody for 45 minutes, washed and mounted in Vectashield mounting medium with DAPI (Vector Labs).

### Flow cytometry

Single cell suspensions were prepared and 1 x10^6^ cells incubated for 20 minutes in Fc block (BD Biosciences) prior to 30 minutes in appropriate antibody cocktails before fixing in Cytoperm/Cytofix (BD Biosciences) for 20 minutes. Data acquisition was performed on a LSR-Fortessa (BD Biosciences), and analysed using FlowJo software.

### Quantitative real-time PCR

Quantitative real-time PCR (qPCR) was determined on cDNA from peritoneal macrophages purified by adherence or MACS sorting (F4/80^+^, over 85% purity). RNA was extracted using GenElute Mammalian Total RNA Miniprep Kit (Sigma) and converted to cDNA using Superscript III (Invitrogen). QuantiTect SYBR Green PCR Master Mix (Qiagen) was used for qPCR. Amplification of the target genes *nitric oxide synthase-2 (Nos2)* and *chitinase-like 3/Ym1 (Chi3l3)* was performed under the following conditions: 30 s denaturation at 95°C, 30 s annealing at 60°C and 30s elongation at 72°C for 40 cycles on duplicate samples using a 7500 Fast Real-time PCR system (Applied Biosystems). PCR amplification efficiencies were established for each primer pair [52] and ranged between 1.9 and 2.1. Cycle threshold (Ct) values of target genes were normalised to the geometric mean of housekeeping genes *hypoxanthine guanine phosphoribosyl transferase (Hprt)*, *beta-2 microglobulin (B2m)* and *hydroxymethylbilane synthase (Hmbs)* [53] and calibrated to the median untreated control luciferase samples for relative quantification by the comparative Ct method [54]. Primers were (5´-3´): *B2m* (forward CTCACACTGAATTCACCCCCA, reverse CATGTCTCGATCCCAGTAGACG); *Hmbs* (forward AGGTCCCTGTTCAGCAAGAA, reverse CATTAAGCTGCCGTGCAACA); *Hprt* (forward ACAGGCCAGACTTTGTTGGA, reverse ACTTGCGCTCATCTTAGGCT); *Nos2* (forward CCGGCAAACCCAAGGTCTAC, reverse CTGCTCCTCGCTCAAGTTCA); *Chi3l3* (forward AAGTTGAAGGCTCAGTGGCT, reverse GTAGATGTCAGAGGGAAATGTCT).

### Cytotoxicity assays

Macrophages were isolated from the peritoneum of mice infected with Tm-sAChE or Tm-luc by MACS sorting (F4/80^+^) on d14 post-infection, and incubated at 5 x 10^5^ ml^-1^ with wild type *T*. *musculi* at 2.5 x 10^6^ ml^-1^ in 96 well plates and cultured for 24 hrs. Serum was isolated from mice 14 days post-infection with *T*. *musculi*-luc or *T*. *musculi*-sAChE, and used at a 1:20 final dilution. Parasite survival was determined by counting motile trypanosomes, and viability further determined by ATP assay, using the CellTiter-Glo Luminescent Cell Viability Assay according to manufacturer's instructions (Promega),

### Statistics

Values are expressed as the mean ± SEM, and significant differences were determined using either Mann-Whitney non-parametric t tests or ANOVA with a 95% confidence interval (GraphPad Prism). P values of <0.05 were considered significant. *p<0.05, **p<0.01, ***p<0.001.

### Accession Numbers

*Nippostrongylus brasiliensis* acetylcholinesterase B precursor, mRNA: GenBank AF052508; *Trypanosoma musculi* small subunit ribosomal RNA gene: GenBank AJ223568.

## Supporting Information

S1 Fig Propagation of *T*. *musculi* in vitro and sensitivity to antibiotics. (A). Trypanosomes were cultured in 50% HMI-9 medium with either 50% DMEM or macrophage-conditioned medium (RAW and J774). B) The effect of passage on trypanosome growth, in which cells were diluted to 5 x 10^4^ ml^-1^ after seven days per passage in 50% HMI-9/50% RAW-conditioned medium. Data are expressed as the mean ±SEM, n = 5. (C) Susceptibility to antibiotics: trypanosomes were grown in the presence of phleomycin (25 μg ml^-1^), hygromycin (50 μg ml^-1^), blasticidin (50 μg ml^-1^), puromycin (2 μg ml^-1^), neomycin (20 μg ml^-1^) or without drug. (D) Susceptibility to blasticidin: trypanosomes were grown in the absence or presence of blasticidin at 1, 5, 25 or 50 μg ml^-1^. Data are expressed as the mean ±SEM (n = 5).
(TIF)

S2 Fig Generation of alternative expression cassettes. A: T7 polymerase inserted into the tubulin array (pT7polyNeo); B) eGFP inserted into the SSU rRNA locus in concert with the T7 promoter and terminator (pSSUT7GFP); GFP inserted into the tubulin array (pTubGFP). Expression of eGFP by different cassettes compared in Fig 1B. (D) Expression of AChE B in cytosol (pSSUAChE). (E) Expression of AChE B as secreted protein (pSSUsAChE). NB encodes sequence for signal peptide of BiP.
(TIF)
