# Supplementary figures and images for: Modulation of the Immune Response by Nematode Secreted Acetylcholinesterase Revealed by Heterologous Expression in Trypanosoma musculi
Source: PLoS Pathog. 2016 Nov 1;12(11):e1005998. doi: 10.1371/journal.ppat.1005998 (PMC5089771; doi:10.1371/journal.ppat.1005998)

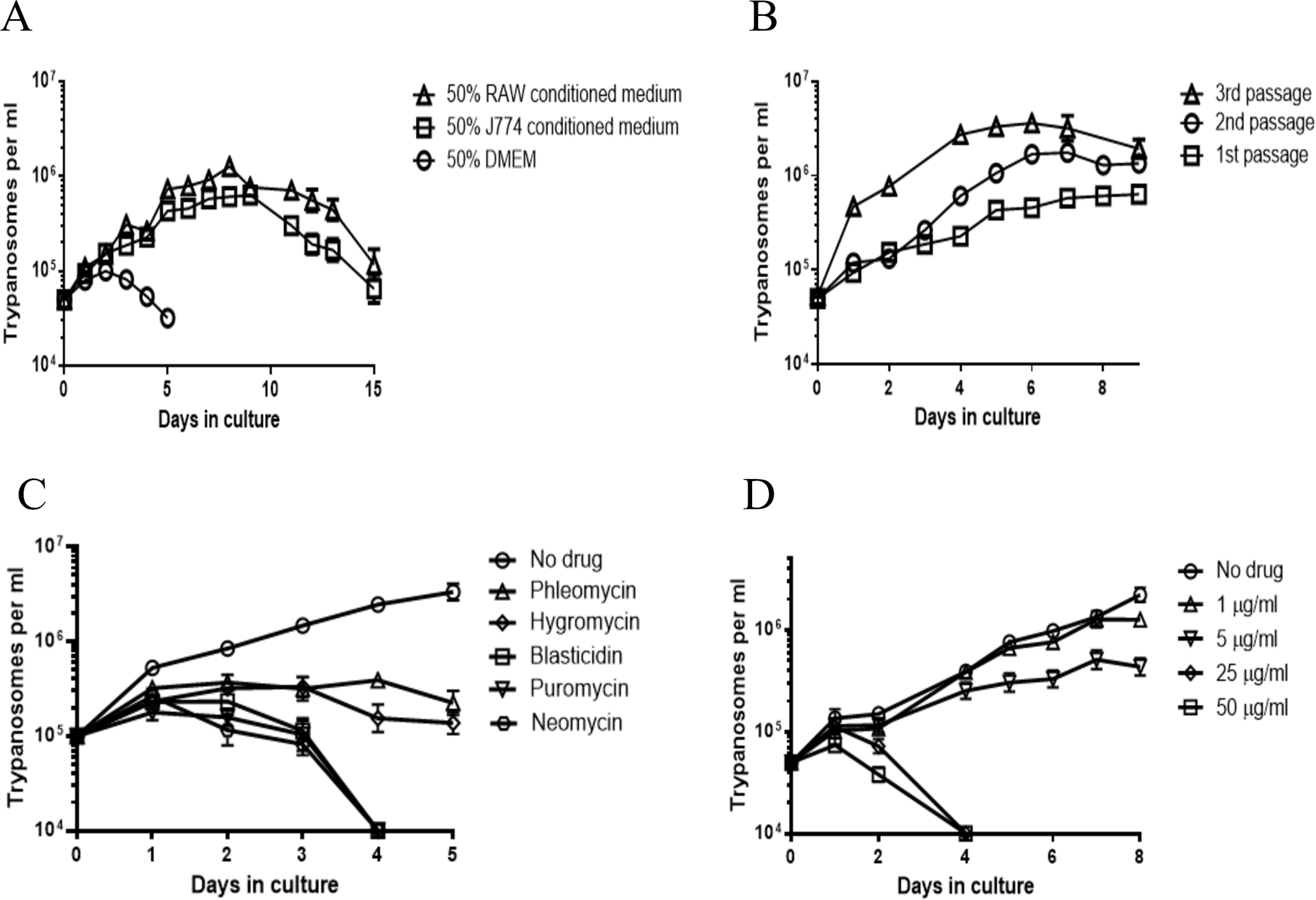

Supplement: S1 Fig — (A). Trypanosomes were cultured in 50% HMI-9 medium with either 50% DMEM or macrophage-conditioned medium (RAW and J774). B) The effect of passage on trypanosome growth, in which cells were diluted to 5 x 104 ml-1 after seven days per passage in 50% HMI-9/50% RAW-conditioned medium. Data are expressed as the mean ±SEM, n = 5. (C) Susceptibility to antibiotics: trypanosomes were grown in the presence of phleomycin (25 μg ml-1), hygromycin (50 μg ml-1), blasticidin (50 μg ml-1), puromycin (2 μg ml-1), neomycin (20 μg ml-1) or without drug. (D) Susceptibility to blasticidin: trypanosomes were grown in the absence or presence of blasticidin at 1, 5, 25 or 50 μg ml-1. Data are expressed as the mean ±SEM (n = 5). (TIF) [file ppat.1005998.s001.tif]

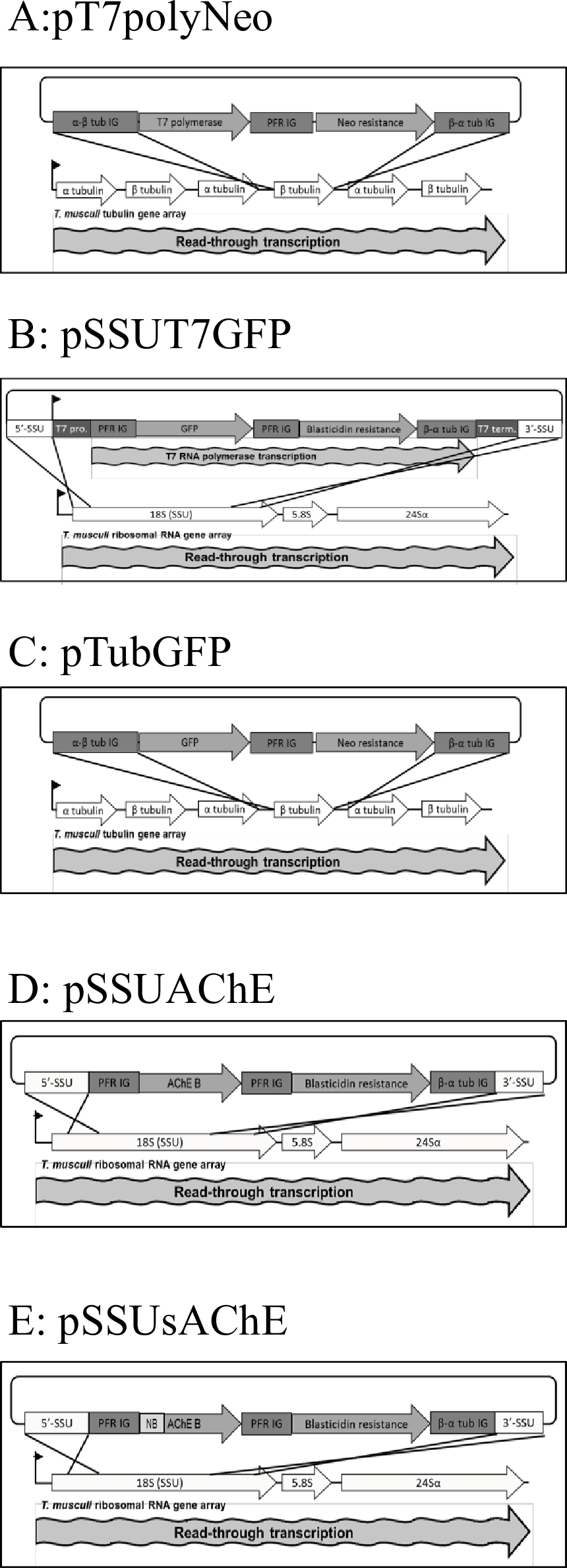

Supplement: S2 Fig — A: T7 polymerase inserted into the tubulin array (pT7polyNeo); B) eGFP inserted into the SSU rRNA locus in concert with the T7 promoter and terminator (pSSUT7GFP); GFP inserted into the tubulin array (pTubGFP). Expression of eGFP by different cassettes compared in Fig 1B. (D) Expression of AChE B in cytosol (pSSUAChE). (E) Expression of AChE B as secreted protein (pSSUsAChE). NB encodes sequence for signal peptide of BiP. (TIF) [file ppat.1005998.s002.tif]
